# Hemicrania continua and unilateral headaches: are they still together in the IHS classification?

**DOI:** 10.1186/1129-2377-16-S1-A35

**Published:** 2015-09-28

**Authors:** Fabio Antonaci, Ottar Sjaastad, Torbjorn A Fredriksen

**Affiliations:** Headache Centre, C. Mondino National Institute of Neurology Foundation, IRCCS, Department of Brain and Behavioral Sciences, University of Pavia, Pavia, Italy; Department of Neurology, St. Olavs Hospital, Trondheim University Hospitals, NTNU, Trondheim, Norway; Department of Neurosurgery, St. Olavs Hospital, Trondheim University Hospital, Trondheim, Norway

Sjaastad & Spierings described “Hemicrania continua” (HC) in 1984[1]. In 2001, succinct criteria were presented[2]: permanent hemicrania, pain intensity: mild-moderate, (but occasionally - severe) and indomethacin dosage < 150 mg daily. In addition, relative shortage of “local” autonomic phenomena, relative lack of “migraine symptoms” and of “cervicogenic” features. Such patients generally had tried legion drugs, with little effect. Such trials equal the usage of placebo. These guidelines seemed to function close to optimally. Then, criteria of the International Headache Society (IHS) (ICHD-III beta classification) came along. Surprisingly, they were transferred from a recent review article by Goadsby[3], almost word by word, despite the existence of a committee of intelligent and knowledgeable colleagues. There is an abundance of failures in the actual scheme. It is unacceptable to include as mandatory criteria, facial/forehead autonomic features. In this way, e.g. sweating becomes prominent - 33%, against a subjective feeling of sweating in only 6% of our series (ratio: 5.5). Objectively, by quantitative evaporimetry, there was *no facial* asymmetry in all our 8 cases. There were 12 autonomic phenomena in this category[3], with a mean ratio between Goadsby's/our figures of 4.4. When made mandatory, autonomic features will create bogus cases. Bogus cases necessitate ultra-high indomethacin dosages; such dosages have an *unspecific*, analgesic effect, on various headaches. Our mean indomethacin continuation dosage was: 83 mg (range: 50-150), while in Goadsby's series it was 176 mg (25-500). HC is the unilateral headache with the least “local” autonomic features, “migrainous” and “vascular” components. It is a rather “pure” headache. The present classification brings HC nearer to other unilateral headaches with local autonomic symptoms, a misunderstood policy. CPH is exceptional with clinical similarities; the absolute indomethacin effect suggests a shared, core pathogenesis.
